# mRNA quality control goes transcriptional

**DOI:** 10.1042/BST20130202

**Published:** 2013-11-20

**Authors:** Cornelia Kilchert, Lidia Vasiljeva

**Affiliations:** *Department of Biochemistry, University of Oxford, Oxford, OX1 3QU, U.K.

**Keywords:** exosome, mRNA, processing, quality control, transcription, Xrn2, CUT, cryptic unstable transcript, DSR, determinant of selective removal, H3K9me, methylated Lys^9^ on histone H3, lncRNA, long non-coding RNA, m^7^G, 7-methylguanosine, PAS, polyadenylation site, polII, RNA polymerase II, PROMPT, promoter upstream transcript, QC, quality control, snoRNA, small nucleolar RNA, snRNA, small nuclear RNA, TRAMP, Trf4–Air2–Mtr4p polyadenylation

## Abstract

Eukaryotic mRNAs are extensively processed to generate functional transcripts, which are 5′ capped, spliced and 3′ polyadenylated. Accumulation of unprocessed (aberrant) mRNAs can be deleterious for the cell, hence processing fidelity is closely monitored by QC (quality control) mechanisms that identify erroneous transcripts and initiate their selective removal. Nucleases including Xrn2/Rat1 and the nuclear exosome have been shown to play an important role in the turnover of aberrant mRNAs. Recently, with the growing appreciation that mRNA processing occurs concomitantly with polII (RNA polymerase II) transcription, it has become evident that QC acts at the transcriptional level in addition to degrading aberrant RNAs. In the present review, we discuss mechanisms that allow cells to co-transcriptionally initiate the removal of RNAs as well as down-regulate transcription of transcripts where processing repeatedly fails.

## Introduction

Most of the steps required to mature a nascent transcript to a functional mRNA take place while the transcript is still attached to elongating polymerase. This has been shown for mRNA capping [1–4], splicing [5–8] and 3′-end formation [9–13], as well as packaging and export [14–16]. All of these different processing steps are heavily interconnected. The cross-talk between processing and the transcription machinery has been discussed in several excellent reviews [17–20]. In the present article, we focus on reviewing recent data linking processing failure to transcriptional regulation.

## QC (quality control) players

The prominent role of nucleases in removing aberrant mRNA species became evident after transcriptome analysis in various QC mutants [21–30]. A central player in nuclear mRNA surveillance is the exosome, a conserved multi-subunit complex that has 3′–5′ exo- and endo-nucleolytic activities. Its exonucleolytic activity resides in two associated enzymes, Dis3 and Rrp6/PM-Scl100; the latter can also function independently of the exosome core [31]. Purified exosome complex is relatively inactive *in vitro* [32] and requires co-factors for full activity, which also play an important role in substrate selection. A well-studied example is the *Saccharomyces cerevisiae* TRAMP (Trf4–Air2–Mtr4p polyadenylation) complex, which contains the poly(A) polymerases Trf4p or Trf5p, the RNA-binding proteins Air1p or Air2p, and the helicase Mtr4p. Taken together, these proteins add short poly(A) tails to RNA substrates, which is thought to make them more accessible [33]. TRAMP also increases the hydrolytic activity of Rrp6p through protein–protein interactions [34].

The role of the exosome in post-transcriptional QC is well established [24,28,29,35,36]. Interestingly, previous studies suggest that it can also act co-transcriptionally. First, there is extensive evidence that Rrp6 and the exosome core are recruited to transcribed genes [37–41]. Secondly, the exosome and its cofactors were shown to directly interact with the processing machinery, including human spliceosome components, *S. cerevisiae* poly(A) polymerase and the SR (serine/arginine-rich) protein Npl3p (Srp2 in *Schizosaccharomyces pombe*) [42–45]. In addition, both *in vivo* and *in vitro* experiments indicate that Rrp6 is required for efficient transcription elongation [46,47]. Finally, computational analysis of gene expression data grouped *rrp6* in a cluster with transcription mutants rather than other decay factors [48], indicating that it may have a function beyond post-transcriptional RNA turnover.

**Figure 1:**
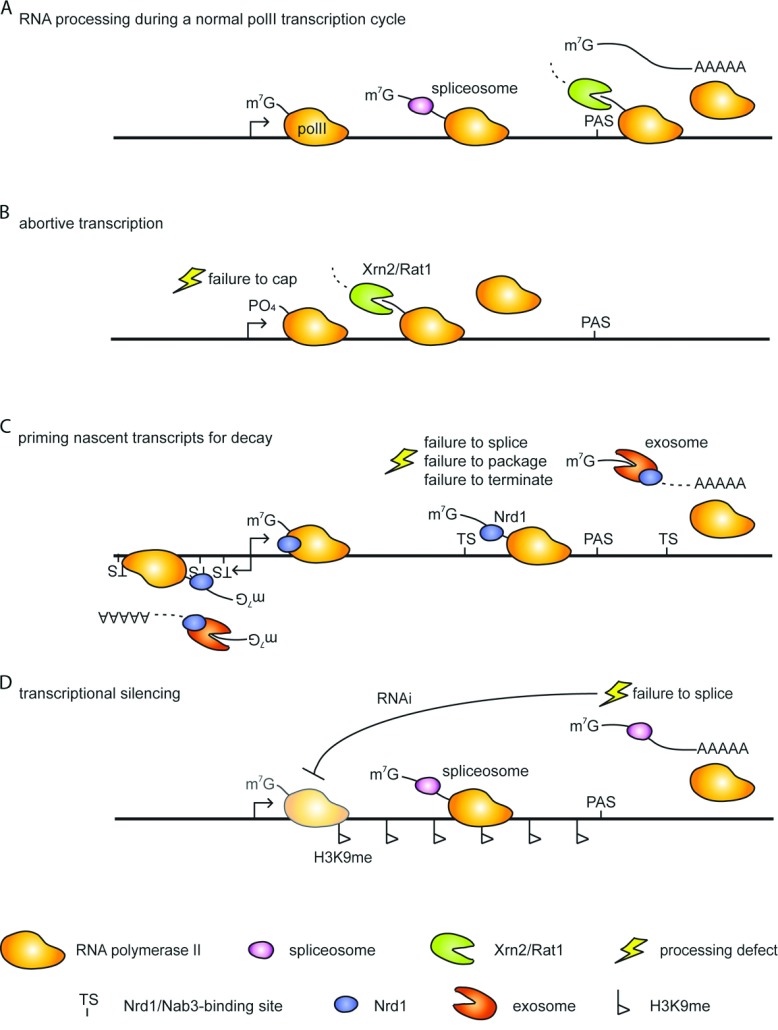
Overview of co-transcriptional QC mechanisms (**A**) RNA processing during the normal polII transcription cycle. The cap structure (m^7^G) is added and introns are removed by the spliceosome. After cleavage at the PAS, the polyadenylated transcript is released and Xrn2 helps to displace polII from the DNA strand. (**B**) Capping defects lead to abortive transcription. Failure to incorporate the correct cap structure results in early recruitment of Xrn2 and premature transcription termination. The nascent RNA is degraded during this process. (**C**) The Nrd1/Nab3 pathway primes nascent transcripts for decay. Sequences enriched in termination sites (TS) are recognized by the Nrd1–Nab3 complex. Initiating polII terminates early and resulting short RNAs (CUTs) are rapidly degraded by the exosome complex. Aberrant RNAs also expose Nrd1–Nab3-binding sites and are rapidly turned over by the exosome complex. (**D**) Inefficiently spliced transcripts induce transcriptional silencing. Stalled spliceosomes recruit the RNAi machinery to poorly spliced transcripts. Transcripts are processed to siRNAs, which lead to deposition of H3K9me and heterochromatin formation.

The 5′–3′ exonuclease Xrn2 (or Rat1p in *S. cerevisiae*), together with its co-factor Rai1, is also involved in nuclear RNA decay and degrades 5′-monophosphorylated substrates [49]. It is also known for its specialized role in transcription termination at the 3′-end of genes (Figure 1A). The mechanism involves the degradation of the nascent transcript by Xrn2 following endonucleolytic cleavage at the PAS (polyadenylation site). In the proposed ‘torpedo’ model, Xrn2-dependent degradation is faster than RNA synthesis, resulting in polII (RNA polymerase II) release from the DNA template [50–53].

## Processing defects and abortive transcription

Addition of the m^7^G (7-methylguanosine) cap occurs shortly after transcription initiation. The structure usually protects mRNAs from degradation and is required for efficient export and translation into protein [54]. Previous studies in budding yeast showed that production of uncapped transcripts caused by mutations that inactivate the capping machinery can lead to co-transcriptional recruitment of Xrn2 and premature transcription termination [55] (Figure 1B). Similarly, abortive transcription was reported to occur when unmethylated cap structures are introduced [56]. These faulty cap structures can be removed by the decapping endonuclease Rai1, resulting in a 5′-monophosphorylated transcript that is susceptible to degradation by Xrn2 [56]. Rai1 can also convert 5′-triphosphorylated RNAs into Xrn2 substrates [57]. In human cells, failure to acquire the correct cap structure leads to decapping and 5′–3′ degradation by the decapping exonuclease DXO (also known as Dom3z; dom-3 homologue Z) [58]. Whether DXO can act co-transcriptionally to promote premature transcription termination of aberrant transcripts is currently not known.

In addition to uncapped mRNAs, aberrant transcripts produced when either splicing or 3′-end formation are impaired can be eliminated co-transcriptionally by Xrn2 [59]. How Xrn2 gains access to these transcripts has not been thoroughly addressed, although decapping by Dcp1 partly accounts for it [59]. Other possible mechanisms that could provide entry for Xrn2 have been discussed, e.g. transcript cleavage at early cryptic PAS or endonucleolytic cleavage by the exosome complex [59]. Another source of endonucleolytic cleavage could be Drosha, which is usually involved in microRNA processing. Drosha has been shown to promote premature termination by Xrn2 to silence retroviruses and transposons [60]. Also, defective downstream processing events such as splicing and 3′-end cleavage could inhibit capping, leading to degradation of the nascent transcript by Xrn2.

All of the examples described above rely on the ability of Xrn2 to down-regulate polII occupancy. At this stage, it is not clear whether Xrn2 simply recognizes the free 5′-monophosphate of the cleaved RNA product or whether its recruitment is facilitated by other means. Cleavage by ribozymes is not sufficient to induce Xrn2-dependent termination [61,62]; however, the products lack the 5′-phosphate, and may represent poor substrates [63,64]. Further research will shed light on this matter.

Premature termination by Xrn2 is also employed to regulate gene expression in a context of functional RNA processing. In *S. cerevisiae*, levels of several lncRNAs (long non-coding RNAs) that govern the expression of inducible genes are regulated by Dcp2 and Xrn2 [65]. In human cells, an Xrn2-dependent mechanism was shown to regulate promoter-proximal pausing and inhibit productive polII elongation, suggesting that there may be a considerable background level of decapping activity [52]. Promoter-proximal pausing is associated with the production of short RNAs from both DNA strands; these RNAs are dependent on Xrn2 and have been suggested to represent fragments protected by stalled polymerase [66,67].

## Priming nascent transcripts for decay

Previous studies have demonstrated that eukaryotic promoters possess an intrinsic bidirectionality, but only transcription in one direction is productive, whereas PROMPTs (promoter upstream transcripts) are rapidly degraded [23,25,26,66,68]. How this directionality is achieved is subject to intense research in diverse species. Recent transcriptome analyses revealed that the regions upstream of human bi-directional promoters are markedly enriched in PASs [69,70]. Interestingly, in contrast with PASs involved in mRNA 3′-end formation, which direct the generation of stable functional transcripts, usage of promoter-proximal PASs is coupled with degradation by the exosome complex. To date, the molecular signal that primes these early-terminating nascent transcripts for decay remains unknown. However, 5′ splice sites appear to prevent the usage of promoter-proximal PASs, and were found to be enriched downstream of promoters [69–71], thereby imposing directionality for productive elongation. In addition to premature transcription termination, histone deacetylation and polII phosphorylation have been implicated in determining promoter directionality in budding yeast [8,72].

Budding yeast has evolved a different, but surprisingly analogous, solution to control productive elongation. Here, promoter-associated CUTs (cryptic unstable transcripts) are terminated by a specialized pathway, which involves the RNA-binding proteins Nrd1 and Nab3 [73,74] (Figure 1C). The Nrd1/Nab3 pathway is also involved in the termination of snRNAs (small nuclear RNAs)/snoRNAs (small nucleolar RNAs) and telomerase RNA, and is coupled to TRAMP-mediated turnover by the exosome complex, or in the case of snRNAs/snoRNAs and telomerase RNA, to exosome-dependent trimming [22,73–80]. Analogous to the enrichment of promoter-proximal PAS upstream of human promoters, the Nrd1–Nab3 complex regulates transcription in response to the ‘quality’ of the DNA template. Although Nrd1–Nab3-binding sites are generally relatively abundant, they are enriched in regions upstream of promoters that give rise to CUTs [81,82]. To induce termination, Nrd1 needs to associate with initiating polII, such that the Nrd1–Nab3 complex is more efficient at terminating short transcripts than longer ones [83,84]. However, increased recruitment of Nrd1 during transcription, even if it does not induce termination, can destabilize a transcript [84,85]. Interestingly, recent studies demonstrated that degradation intermediates originating from unspliced RNA species can be UV-cross-linked to Nrd1, Nab3 or Trf4 [82], suggesting that aberrant RNAs are also primed for decay via this pathway (Figure 1C). In *S. cerevisiae*, Nrd1 has been found genome-wide at introns, potentially recruiting the exosome complex to unspliced RNAs [86]. Also, Nrd1–Nab3-dependent termination was shown to provide a failsafe for transcripts that read past a PAS, thereby restricting mRNAs where 3′-end formation has failed [61,75]. How Nrd1 is recruited to aberrant RNAs is not understood; however, it is possible that Nrd1 is targeted to RNAs that are not properly packaged. For example, studies of genes involved in the heat-shock response in budding yeast revealed that defective packaging on mutation of the THO complex leads to recruitment of RNA decay factors and degradation of these RNAs [87,88]. RNA destabilization can be reversed by deletion of Rrp6 or Trf4 [87,89]. Interestingly, TRAMP and the exosome appear to influence the levels of polyadenylation factor Fip1, thereby down-regulating canonical polyadenylation in THO mutants [90]. Although this effect is global in THO mutants, it is of course very tempting to speculate that this mechanism can act locally on aberrant mRNAs, and specifically target these for degradation.

It is not clear whether there are functional homologues of Nrd1 and Nab3 in other species. Different proteins may be used for a similar purpose, e.g. in *Drosophila*, Su(s) (suppressor of sable) co-transcriptionally recruits Rrp6 to aberrant RNAs that contain transposable elements [91]. The TRAMP complex, however, is conserved from *S. pombe* to flies and mammals, and with it the principle of a degradative polyadenylation, although the range of substrates differs between species [33]. Although the coupling of Nrd1–Nab3-dependent termination with RNA destabilization in *S. cerevisiae* is quite well understood, it is less clear how substrates are selected for degradation in other organisms. For example, the TRAMP-mediated destabilization of RNAs produced from heterochromatic regions in fission yeast appears to be sequence-independent [92]. Here, RNA destabilization contributes to heterochromatic gene silencing in parallel to RNAi [93]. RNAi directs the deposition of H3K9me (methylated Lys^9^ on histone H3) that recruit chromodomain protein 1 (Swi6/HP1) leading to heterochromatin formation. Both H3K9me and recruitment of Swi6 are required for rapid heterochromatic RNA turnover, but not sufficient to elicit decay on other RNAs [24,93,94]. In addition to heterochromatic regions such as centromeres and telomeres, H3K9me can be found at selected genes, such as those encoding meiotic RNAs, which are repressed by RNAi and the exosome in mitotic cells [95,96]. In contrast with RNAs derived from heterochromatic regions, meiotic RNAs are targeted by these machineries in a sequence-specific manner. This involves the RNA-binding protein Mmi1 that recognizes so-called DSRs (determinants of selective removal) on these transcripts and is believed to facilitate the recruitment of both RNAi and exosome [96–98].

## QC and transcriptional silencing

Besides the mechanisms outlined above, loci encoding RNAs that are intrinsically poorly processed, e.g. due to suboptimally positioned regulatory elements, can be targeted by another mechanism: transcriptional silencing. This was demonstrated for poorly spliced genes in *Cryptococcus neoformans* [99]. Here, inefficiently spliced introns cause the retention of stalled spliceosomes on a transcript. The spliceosome then recruits the RNAi machinery, and processing of splicing intermediates into siRNAs leads to heterochromatin formation across the gene (Figure 1D). Recruitment of the spliceosome had been shown previously to facilitate RNAi-dependent heterochromatin formation in *S. pombe* [100], indicating that the pathway may be conserved. Another example of transcriptional silencing initiated in response to processing defects was reported for convergent genes in *S. pombe*, where failure to terminate can produce overlapping read-through transcripts [101]. These transcripts form dsRNAs, which are processed into siRNAs by the RNAi machinery and act to silence transcription.

To date, studies supporting a role for transcriptional silencing in mRNA QC have mostly been carried out in yeasts. However, comparable mechanisms may exist in metazoans. One report links exosome-dependent retention of unspliced pre-mRNAs to transcriptional silencing at the locus in *Drosophila* [102]. Interestingly, nonsense-mediated transcriptional silencing, which is observed in response to the introduction of premature stop codons into human immunoglobulin minigenes [103], was also linked to a retention at the site of transcription, combined with a splicing defect [104]. Other examples are sure to follow.

## Conclusion

It has become clear that cells do not only rely on their capacity to identify and clear up dysfunctional RNAs after they have been released into the nucleoplasm, but that mechanisms are in place that respond to processing errors while the transcript is still attached to chromatin. This has a dual advantage for the cell; first, it provides additional checkpoints for the removal of unprocessed and potentially deleterious transcripts, which are less likely to escape post-transcriptional RNA surveillance. Secondly, resources are mainly used to make correct RNA, thus avoiding wasteful transcription. Many of the important players in co-transcriptional QC, such as the exosome and Xrn2/Rat1, are widely conserved; however, their importance in dealing with different classes of substrates can vary between species (Table 1). There are a number of important questions that remain to be answered. How are specific substrates selected for co-transcriptional QC? What molecular signals prime different aberrant RNAs for exosome-dependent turnover? What prevents QC systems from targeting properly processed functional mRNAs? Future research will hopefully shed light on these issues.

**Table 1:** Reported substrates of co-transcriptional QC mechanisms

Defect/affected process	Organism	How defect was introduced	Reference(s)
Abortive transcription			
Uncapped	*S. cerevisiae*	*ceg1-63*	[55]
Unmethylated cap	*S. cerevisiae*	*abd1-5*	[56]
Triphosphorylated cap	*S. cerevisiae*	–	[57]
lncRNAs	*S. cerevisiae*	–	[65]
Failed splicing	Human	Mutated 3′ splice site	[59]
Failed 3′-end formation	Human	Mutated PAS	[59]
Cleaved transcript	Human	–	[60]
Paused polymerase	Human	–	[52]
Priming nascent transcripts for decay			
PROMPTs	Human	–	[69,70]
CUTs	*S. cerevisiae*	–	[73,74]
Unpackaged RNA	*S. cerevisiae*	+Rho/THO mutants	[85,87,89]
Failed splicing	*S. cerevisiae*	–	[82]
Failed 3′-end formation	*S. cerevisiae*	–	[61]
Heterochromatic RNA	*S. cerevisiae*	–	[105,106]
Heterochromatic RNA	*S. pombe*	–	[93]
DSR-containing RNAs	*S. pombe*	–	[97]
Transposable elements	*Drosophila*	–	[91]
Transcriptional silencing			
Failed splicing	*C. neoformans*	–	[99]
Failed splicing	*Drosophila*	Mutated 5′/3′ splice site	[102]
Failed splicing	*S. pombe*	–	[100]
Failed 3′-end formation	*S. pombe*	–	[101]
DSR-containing RNAs	*S. pombe*	–	[96,98]
Premature stop codon	Human	–	[103,104]
